# Institutional Notes and News

**Published:** 1910-10-01

**Authors:** 


### Institutional Notes and News.

**Lady Ridgeway Memorial Hospital, Colombo.**

The Lady Ridgeway Memorial Children's Hospital has, we learn from the *Times* correspondent, been opened at Colombo, Ceylon. The ceremony was performed by the Governor, Sir Henry McCallum, who announced that a telegram from Sir West Ridgeway had been received which expressed the heartfelt gratitude he felt that the memorial to his wife had been completed. The building, which has been raised to adjoin the Lady Havelock Women's Hospital, has been constructed at a cost of 52,000 rupees, which are equivalent to £3,466 of English money. We congratulate the citizens of Colombo on the form which the memorial has taken, and appreciate the satisfaction which they must feel at having a new children's hospital. That the hospitals of Colombo should be identified with the honoured names of Lady Havelock and Lady Ridgeway is a tribute to the spirit in which hospital work is regarded in the city, and we feel sure that the two ladies themselves would have felt an honourable pride that the treatment of women and children should be associated thus intimately with their names.

**Middlesex Hospital.**

Figures do not, as a rule, preponderate in this column, but our readers may be interested in a brief analysis of the subscription list by which the £20,000 has just been raised for the Middlesex Hospital. This analysis will show on what class of gifts a large public subscription depends. Mr. F. Clare Melhado has supplied the figures. Between the two extremes of one thousand guineas and three pence, we find that five shillings was subscribed by the largest number of people, and that the twenty-six persons who gave a sum over £100 and less than £250 contributed the largest sum of any group of subscribers. They were, however, hard pressed by the seven princely donors of £500, and by the thirty hardly less princely persons who gave a hundred pounds apiece. The givers of ten pounds were the group next in order of productivity, while no less than some forty pounds was realised by the generosity of those who could afford less than five shillings. It will be seen, then, that the value of the small subscription is proved no less when a large sum is aimed at than when a small one is wanted. In this instance we see that every class seems to have come forward in a representative manner, and each will feel that its efforts have been rewarded by the triumphant result of the appeal. Annual subscribers must now come forward to keep the hospital as free of liability as it stands to-day.

**West of England Eye Infirmary.**

Last week the third and final phase in the extension and development of the West of England Eye Infirmary was completed by the formal opening of the Centenary Wing. The hospital was founded in 1808, and as far back as 1895 a scheme was prepared by which it was hoped worthily to commemorate the institution's hundredth birthday. Sir Bramwell Thomas made the designs, and his great work was concluded only the other day, when he presented the gold key to the Lord Lieutenant of Devon, Earl Fortescue, for the opening ceremony. Some idea of the architectural scale on which the enlarged infirmary has been planned is gained by recalling the cost of each section of the building operations. The first two portions were the most expensive of the three, each costing £8,500, while that now finished cost £4,000. The West of England—for it is not Exeter nor Devon alone which sends patients to the Eye Infirmary—has now a very magnificent institution, the result of much forethought, revision, and generosity. The year 1908 has been commemorated worthily, and we wish the institution many happy returns of its centenary.

